# Relation of Leukocytes and Its Subsets Counts with the Severity of Stable Coronary Artery Disease in Patients with Diabetic Mellitus

**DOI:** 10.1371/journal.pone.0090663

**Published:** 2014-03-05

**Authors:** Li-Feng Hong, Xiao-Lin Li, Song-Hui Luo, Yuan-Lin Guo, Jun Liu, Cheng-Gang Zhu, Ping Qing, Rui-Xia Xu, Na-Qiong Wu, Li-Xin Jiang, Jian-Jun Li

**Affiliations:** 1 Division of Dyslipidemia, State Key Laboratory of Cardiovascular Disease, Fu Wai Hospital, National Center for Cardiovascular Diseases, Chinese Academy of Medical Sciences, Peking Union Medical College, Beijing, China; 2 Division of Cardiology, The Fifth Hospital of Wuhan & Affiliated Guangci Hospital of Wuhan University, Wuhan, China; Children's Hospital Boston, United States of America

## Abstract

**Background:**

Both coronary artery disease (CAD) and diabetes mellitus (DM) are associated with inflammation. However, whether and which leukocytes can predict the presence and extent of CAD in patients with DM has not been investigated. The aim of the present study was to examine the association of leukocyte and its subsets counts with the severity of CAD in patients with DM.

**Methods and Findings:**

Three hundred and seventy-three diabetic patients who were scheduled for coronary angiography due to typical stable angina pectoris were enrolled in this study. They were classified into the three groups according to tertiles of Gensini score (GS, low group <8, n = 143; intermediate group 8∼28, n = 109; high group >28, n = 121). The relationship between the leukocyte and its subsets counts with the severity of CAD were evaluated. The data indicated that there were significant correlations between leukocyte and neutrophil counts with GS (r = 0.154 and 0.156, respectively, all P<0.003 for Pearson's correlation). Similarly, area under the receivers operating characteristic curve of leukocyte and neutrophil counts were 0.61 and 0.60 respectively (95%CI: 0.55–0.67, all P = 0.001) for predicting high GS. Multivariate logistic regression analysis demonstrated that leukocyte count was an independent predictor for high GS patients with DM (OR = 1.20, 95%CI 1.03–1.39, P = 0.023) after adjusting for conventional risk factors of CAD.

**Conclusions:**

Compared with its subsets, leukocyte count appeared to be an independent predictor for the severity of CAD and the optimal cut-off value for predicting high GS (>28 points) was 5.0×10^9^ cells/L in diabetic patients.

## Introduction

Since low grade of local and systemic inflammation is characteristic of all stages of atherosclerosis, multiple markers of inflammation have been intensively evaluated as potential risk factors for the development of coronary artery disease (CAD) and its complications, such as high-sensitivity C-reactive protein (hs-CRP), interleukin-6, fibrinogen, leukocyte and its subsets counts [1]–[6]. Previous studies have provided strong evidences of association between the frequency of leukocytes, the frequency of leukocyte subsets or the ratio of neutrophil/lymphocyte with CAD [7]–[12]. Moreover, some of these studies clearly reported a positive correlation between the frequency of circulating leukocytes or leukocyte subsets with adverse outcome in CAD patients or in apparently healthy individuals with perivascular disease or in patients with heart failure [7], [9], [10], [13]–[17]. Further, a few studies demonstrated the relationship between leukocyte count and presence, severity and progression of the atherosclerotic plaque in patients with either acute coronary events or stable CAD [8], [12], [18]–[22]. On the other side, in patients with moderate and high-risk of non-ST-segment elevation acute coronary syndrome (ACS), increased leukocyte count at admission in the clinic was an independent predictor of major bleeding at 30 days, or mortality at 1 year [14]. Interestingly, a study indicated that the leukocyte count was qualified to predict myocardial infarct size whereas CRP was not in patients with ST-segment elevated myocardial infarction who had been treated with primary percutaneous coronary intervention [23]. Based on these studies, high leukocyte and its subsets counts, even within the normal range, appeared to be not only linked to systemic inflammatory response but also to increased risk of cardiovascular disease and adverse prognosis.

Although leukocyte count greater than 6.7∼6.9×10^9^ cells/L may identify individuals at high-risk of CAD, current clinical practice does not consider it a useful predictor of CAD [7], [24]–[26]. Moreover, there is not robust consensus in the clinical practice on the leukocyte range association with CAD [16], [27]. This may be due to a wide range of frequency in subjects at high risk, to the investigated population or to unknown confounding factors [24]. Therefore, there is still a need to investigate the association between the frequency of leukocyte subsets and CAD, in subjects with a different disease status.

Furthermore, it is still unknown whether the frequency of leukocytes or of a specific leukocyte subset can be a useful predictor of CAD onset and of its severity. In the present study, we hence prospectively assessed the correlation of leukocyte and its subsets counts with the severity of CAD by Gensini Score (GS) in patients with type 2 diabetic mellitus (DM) who underwent coronary angiography.

## Research Design and Methods

### Study design and population

The study complied with the Declaration of Helsinki, and was approved by the hospital ethical review board (Fu Wai Hospital & National Center for Cardiovascular Diseases, Beijing, China). Informed written consent was obtained from all patients included in this analysis.

From June 2011 through March 2012, we prospectively enrolled 373 type 2 diabetic patients (men: 70.2%) aged 31 to 79 years (average age 58.7 years) who had a typical stable exertional angina pectoris and was referred for selective coronary angiography to our center. Patients with type 1 diabetes mellitus, ACS, significant hematologic disorders (leukocytes count ≤3.5×10^9^ cells/L or ≥20×10^9^ cells/L), infectious or inflammatory disease, and severe liver and/or renal insufficiency were excluded from the current study. All subjects enrolled in this study underwent detailed clinical, hematologic and angiographic examination for assessment of the cardiac status. Demographic data and history of exposure to risk factors for CAD, such as smoking habits, hypertension, hyperlipidemia, obesity, DM, previous stroke, peripheral vascular disease, family history of CAD and non-cardiovascular diseases were also collected.

Hypertension was diagnosed when repeated blood pressure measurements were ≥140/90 mmHg (at least two times in different environments) or if the patient was taking anti-hypertensive drugs. DM was diagnosed in patients with fasting serum glucose level ≥6.99 mmol/L in multiple determinations or in patients under active treatment with insulin or oral hypoglycemic agents. Hyperlipidemia was considered to be present in patients with fasting total cholesterol (TC) ≥200 mg/dl or TG ≥150 mg/dl. CAD was identified in the presence of obstructive stenosis in at least 50% of the vessel lumen diameter in any of the main coronary arteries according to the diagnosis of two independent senior interventional cardiologists who performed quantitative coronary angiography (QCA) analysis. The severity of CAD was represented by GS system [28]. The left ventricular ejection fraction (LVEF) was evaluated by echocardiograph using the area-length methods with modified Simpson's rule [29].

### Biomarker measurements

Venous blood samples were obtained from each patient at baseline upon admission. The levels of leukocytes, neutrophils, lymphocytes, and monocytes were determined using an automated blood cell coulter by a Coulter LH780 Hematology Analyzer (Beckman Coulter Ireland Inc Mervue, Galway, Ireland). The levels of hemoglobin A1c (HbA1c) were measured using the Tosoh G7 Automate HPLC Analyzer (TOSOH Bioscience, Japan). The concentrations of hs-CRP were determined using immunoturbidimetry (Beckmann Assay 360, Bera, Calif., USA). Total cholesterol (TC) and triglyceride (TG) were measured by enzymatic methods and high-density lipoprotein cholesterol (HDL-C) by a direct method (Roche Diagnostics, Basel, Switzerland). Low-density lipoprotein cholesterol (LDL-C) was obtained by Friedewald's formula (if fasting triglycerides <3.39 mmol/l) or by ultracentrifugation. ApoB was measured by an immunoturbidimetric method (Tina-quant, Roche Diagnostics) calibrated against the World Health Organization/International Federation of Clinical Chemistry reference standard SP3–07. All other biomarkers were analyzed by standard hematological and biochemical tests.

### Statistical analysis

Quantitative variables were expressed as mean±standard deviation (SD), and qualitative variables were expressed as numbers and percentages. Quantitative and qualitative variables were analyzed by the Kruskal–Wallis one-way analysis of variance, chi-squared statistic tests, or Student's T tests when appropriate. Correlation between variables was examined using Spearman and Pearson correlation coefficient when appropriate. Receiver operating characteristic (ROC) curves were constructed at the most discriminating cut-off point values aiming to document the predictive power of leukocyte and its subsets counts for high GS. Based on the tertiles of GS, the enrolled patients were classified into the three groups (low group<8-point, n = 143; intermediate group 8–28 points, n = 109; high group >28-point, n = 121). Predictive effect of leukocyte and its differential counts for high GS was carried by binary logistic regression models using forward stepwise selection process. A p value of less than 0.05 was considered statistically significant. Statistical studies were carried out with the SPSS program (version 19.0, SPSS, Chicago, Illinois, USA).

## Results

### Baseline characteristics

The cohort in the current study consisted of 373 type 2 diabetic patients admitted to the clinic for coronary angiography with an average age of 58.7±9.6 years (median = 59 years; ranged from 31 to 79 years). The mean GS was 22.9±23.2 (median = 14 points; ranged from 0 to 124 points). The baseline demographic, clinical characteristics and laboratory findings of the enrolled subjects by the tertiles of GS (low group <8, n = 143, 38.3%; intermediate group 8∼28, n = 109, 29.2%; high group >28, n = 121, 32.4%) are summarized in Table 1. In brief, patients with higher GS showed a lower LVEF and HDL-C levels, but higher N-terminal pro-brain natriuretic peptide (NT-pro-BNP), HbA1c, and serum creatinine levels. Similarly, there were significant differences in major inflammatory and oxidative stress biomarkers such as plasma fibrinogen, uric acid, and hs-CRP among the groups. More important, the leucocyte and neutrophil counts were significantly different among these groups as assessed by trend analysis and comparison test.

**Table 1:** Baseline demographic, clinical and laboratory characteristics based on tertiles of Gensini score.

Variables	Low (<8;n = 143)	Intermediate (8∼28;n = 109)	High (>28;n = 121)	P-value for trend^a^	P-value^b^
Risk factors					
Age(years)	56.7±9.9	60.0±9.4	59.8±8.9	0.008	0.121
Male gender	94(65.7)	78(71.6)	90(74.4)	0.291	0.226
BMI(kg/m^2^)	25.7±3.3	24.9±2.8	25.7±3.0	0.120	0.447
Current smoking	68(47.6)	59(54.1)	70(57.9)	0.235	0.121
Hypertension	85(59.4)	77(70.6)	82(67.8)	0.145	0.508
Hyperlipidemia	100(69.9)	88(80.7)	99(81.8)	0.039	0.177
PVD	3(2.1)	3(2.8)	2(1.7)	0.847	0.650
Prior Stroke	6(4.2)	3(2.8)	6(5.0)	0.690	0.523
Family history of CAD	7(4.9)	13(11.9)	17(14.0)	0.033	0.064
Laboratory test					
Leukocyte(10^9^/L)	6.3±1.5	6.2±1.6	6.8±1.5	0.003	0.001
Neutrophil (10^9^/L)	3.6±1.2	3.6±1.1	4.0±1.2	0.006	0.001
Lymphocyte(10^9^/L)	1.9±0.6	1.9±0.6	2.0±0.6	0.595	0.308
Monocyte(10^9^/L)	0.5±0.2	0.5±0.2	0.5±0.2	0.544	0.519
N/L ratio	2.1±1.1	2.0±0.8	2.2±1.1	0.393	0.229
hs-CRP (mg/L)	3.1±3.9	2.3±3.5	4.0±4.5	0.006	0.006
FBG (mmol/L)	5.6±1.6	6.4±2.7	6.2±1.9	0.009	0.253
Hemoglobin (g/L)	139.4±15.2	138.3±15.6	137.1±15.6	0.505	0.305
HbA1c (%)	6.4±1.2	6.9±1.6	7.0±1.3	0.000	0.004
Platelet count(10^9^/L)	204.5±60.4	192.0±45.8	206.5±55.4	0.098	0.224
Fibrinogen(g/L)	3.0±0.8	2.9±0.7	3.3±0.9	0.000	0.000
D-dimer (mg/dL)	0.4±0.5	0.4±0.5	0.4±0.6	0.075	0.487
Bilirubin (umol/L)	15.3±5.4	15.1±5.6	15.4±7.4	0.969	0.836
ALP (IU/L)	64.2±17.9	61.6±19.1	62.6±17.4	0.517	0.816
AST(IU/L)	19.4±13.3	18.5±9.2	17.4±10.0	0.342	0.185
ALT (IU/L)	31.2±33.3	29.7±21.9	28.7±25.1	0.772	0.554
Creatinine (umol/L)	73.8±15.0	75.6±16.4	78.6±14.9	0.041	0.019
Uric Acid (mmol/L)	335.6±75.6	323.3±80.8	354.6±77.4	0.009	0.005
NT-pro-BNP(fmol/mL)	661.1±486.8	667.9±485.2	893.5±764.8	0.305	0.000
LVEF (%)	62.8±7.7	63.1±7.4	60.2±9.5	0.014	0.003
Lipid profile					
Triglycerides(mmol/L)	1.7±1.0	1.7±0.8	1.8±1.1	0.434	0.230
TC (mmol/L)	4.0±1.0	4.0±0.9	4.1±1.1	0.572	0.360
LDL-C(mmol/L)	2.3±0.9	2.4±0.8	2.5±0.9	0.292	0.121
HDL-C(mmol/L)	1.1±0.3	1.1±0.3	1.0±0.2	0.011	0.009
Lipoprotein (a) (mg/L)	237.7±217.5	190.9±211.2	289.7±283.6	0.008	0.007
apoA(g/L)	1.4±0.3	1.5±0.3	1.4±0.3	0.012	0.057
apoB(g/L)	1.0±0.3	1.0±0.3	1.1±0.3	0.045	0.015
Prior treatment					
Aspirin	136(95.1)	106(97.2)	118(97.5)	0.501	0.463
Beta-blocker	103(72.0)	87(79.8)	109(90.1)	0.001	0.001
ACE-I/ARB	27(18.9)	22(20.2)	44(36.4)	0.002	0.000
Statin	125(87.4)	109(100)	116(95.9)	0.000	0.258

BMI = Body mass index; PVD = Peripheral vascular disease; CAD = Coronary artery disease; LVFE  =  Left ventricular ejection fraction; NT-pro-BNP = N-terminal pro-Brain natriuretic peptide; hs-CRP = high sensitivity C-reactive protein; N/L ratio  = Neutrophil count to lymphocyte count ratio; HbA1c = Glycosylated hemoglobinA1C; FBG = Fasting blood glucose; ALP = Alkaline phosphatase; AST = Aspartate aminotransferase; ALT = Alanine aminotransferase; TC = Total cholesterol; LDL-C = Low density lipoprotein cholesterol; HDL-C = High density lipoprotein cholesterol; ACE-I = Angiotensin converting enzyme inhibitors; ARB = Angiotensin receptor blocker.

a P-value obtained from analysis of variance, Kruskal-Wallis test, or chi-squared test.

b P-value for high GS versus non-high (low and intermediate) GS.

### Correlation between frequency of leukocytes and HbA1c, hs-CRP or GS

To explore the relationship of leukocytes and other biomarkers in type 2 diabetic patients with CAD, a correlation evaluation was performed in the present study. Using Spearman and Pearson correlation analysis, correlation between the frequency of leukocyte and HbA1c, hs-CRP or GS was reported in Table S1. There was a significant correlation of leukocytes frequency with GS. The same was valid for HbA1c and hs-CRP with leukocyte and neutrophil counts. However, there was no correlation of lymphocyte and monocyte counts with HbA1c, hs-CRP and GS (Table S1).

### Utility of frequency of leukocytes for predicting severity of CAD in diabetic patients

**Figure 1:**
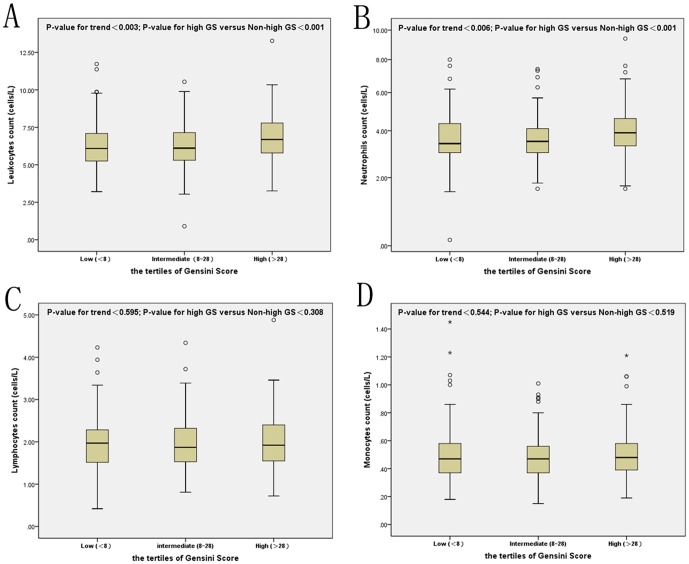
The results of leukocyte (A), neutrophil (B), lymphocyte (C) and monocyte (D) counts according to the Gensini Score.

**Figure 2:**
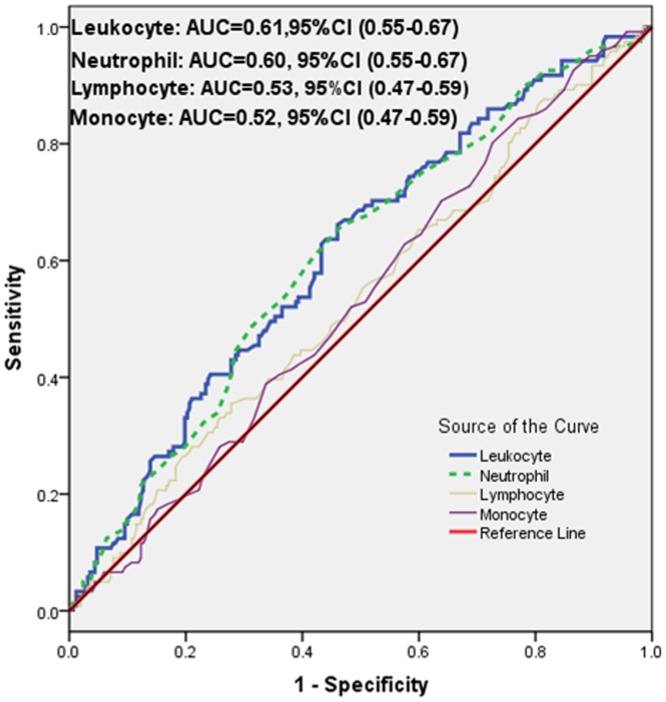
The results of receiver operating characteristic (ROC) curve analysis for the predictive power of the leukocyte counts and the Gensini score.

As shown in figure 1, there was a significant correlation of leukocyte and neutrophil counts with the tertiles of GS but not of lymphocyte or monocyte counts (chi-squared for trend of leukocyte, neutrophil counts, P = 0.003 and P = 0.006, respectively, Figure 1). AUC of leukocyte and neutrophil counts were 0.61 and 0.60 respectively (95%CI 0.55–0.67, P = 0.001 for both) for predicting high GS (Figure 2). The optimal cut-off values of leukocyte and neutrophil counts to predict high GS were 5.0×10^9^ cells/L and 4.5×10^9^ cells/L respectively. Additionally, as presented in table 2, the results of multivariate logistic regression for predicting high GS suggested that only total leukocyte count was an independent predictor of the severity of CAD after adjusting for gender, age, BMI, current smoking, hypertension, hyperlipidemia, peripheral vascular disease, prior stroke, family history of CAD, lipid parameters, serum creatinine, and hs-CRP (OR = 1.20, 95% CI 1.03–1.39, P = 0.023).

**Table 2:** Univariate and multivariate logistic regression analysis to determine the independent predictor of high Gensini Score.

Variables	Univariate		Multivariate	
	O.R.(95%CI)	P-value	O.R.(95%CI)	P-value
Uric acid	1.00(1.00–1.01)	0.006	1.00(1.00–1.01)	0.007
NT-pro-BNP	1.00(1.00–1.00)	0.002	1.00(1.00–1.00)	0.023
Fibrinogen	1.69(1.28–2.23)	0.000	1.42(1.06–1.91)	0.020
HbA1C	1.24(1.07–1.44)	0.005	1.23(1.05–1.45)	0.015
Leukocytes	1.28(1.10–1.47)	0.001	1.20(1.03–1.39)	0.023

NT-pro-BNP = N-terminal pro-Brain natriuretic peptide; HbA1c  =  Glycosylated hemoglobinA1c.

## Discussion

To our knowledge, this was the first study that focused on the association of leukocytes and its subsets counts with the severity of CAD in patients with DM. The main findings of the present study could be summarized in five aspects. First of all, DM patients with high GS (>28 points) showed the lower levels of LVEF and HDL-C but high levels of NT-pro-BNP, HbA1c, fibrinogen, serum creatinine and the inflammatory and oxidative stress biomarkers (leucocytes, neutrophils, uric acid, and hs-CRP). Secondly, in agreement with published studies on non-diabetic population, as showed in ROC curves and box graphs, the data demonstrate that elevated leukocyte and neutrophil counts might be useful discriminators of CVD severity in diabetic patients with stable CAD but not lymphocyte and monocyte counts [8], [16], [18], [20]. Thirdly, we have directly correlated leukocyte and differential counts with GS and other inflammatory markers. Moreover, unlike earlier investigations, our multivariate logistic regression analysis, after adjusting for major potential confounders, found that leukocytes but not neutrophils is an independent predictor for high GS [12], [15]. Finally, although the power of the present study was relatively small, ROC curves showed that leukocyte count >5.0×10^9^ cells/L associates with increased risk of severe CAD in type 2 diabetic population, which is a value much lower than the threshold in the non-diabetic population (6.9×10^9^ cells/L) [16]. Thus, our study might extend the previous study and provide novel findings regarding the role of total leukocytes and its subsets in predicting the risk of cardiovascular disease in diabetic patients.

Numerous studies have validated the pivotal role of inflammation in the pathogenesis of atherosclerosis [30]. Several clinical cohorts, meta-analysis as well as case-control studies have provided compelling evidence that inflammation is involved in both initiation and progression of the atherosclerotic process [26], [27], [31]. Moreover, various triggers of inflammatory responses lead to acute or chronic leukocytosis as well as synthesis of local and systematic non-specific molecules. In this setting, increased leukocyte count probably plays a key role in the reparative process that occurs to replace the necrotic tissue with collagen. In details, leukocytes might be recall in the site of inflammation as a consequence of endothelial cell injury caused by proteolytic enzymes, release of monocytes tissue factors, activation of coagulation system, resulting in increased leukocytes adhesion, damage to the endothelial cells and alteration of the vessel flow [27]. In addition, these effects might vary with the increase level of circulating inflammatory markers in individuals with different risk of developing CAD [19]. It has been demonstrated a positive association between increased leukocyte count, within the “normal” range, or neutrophils/lymphocytes ratio (N/L) and the prevalence or extent of stable CAD and acute myocardial infarction. Furthermore, chronic inflammation presented by increasing leukocytes count within normal range, might play a role in the development of macro- and microvascular complications in diabetic patients [6]. In the present study, we found the positive correlations of high GS with leukocytes and neutrophils, but not with lymphocytes and monocytes. One explanation might be that the leukocytes signal comes from neutrophils, the most abundant population in peripheral blood especially in an acute inflammatory state.

There were three studies that have demonstrated the correlation of leukocyte count with CAD incidence [32]–[34]. Braunwald and colleagues evaluated the relationship between the baseline white blood cell (WBC) and angiographic findings as well as clinical outcomes in 2,208 patients with unstable angina/non-ST-segment elevation ACS [18]. They found that elevated leukocytes count was not only associated with impaired epicardial and myocardial perfusion but also with the extent of CAD and higher mortality. Moreover, after adjustment for typical risk factors and other biomarkers, WBC count and hs-CRP could be used to stratify patients across an eightfold gradation of six-month mortality risk. Data from Rasouli M et al in a small sample size study on stable CAD suggested that the total leukocyte count and its subgroups were associated with the presence and severity of CAD, although this association was not independent from other coronary risk factors [8]. Study performed by Avanzas et al. showed that neutrophil count and hs-CRP level were higher in patients of stable CAD compared to those without. Nevertheless, they detected that neutrophil count but not hs-CRP level was correlated with angiographic stenosis complexity [35]. More recently, a prospective cohort study performed in 3005 patients with coronary angiography assessed the association of N/L ratio with the degree of CAD. They found that N/L ratio was qualified as an independently predictor for the extent of CAD and 3-years outcome using a multivariate regression analysis [20]. Recently, clinical observations from Kaya and Sahin groups evaluated the severity of CAD using Syntax score and led to similar conclusion [21], [36]. Unfortunately, these studies did not focus on diabetic patients.

Therefore, the present work not only confirmed findings of previous studies but also provided novel insights concerning the role of leukocytes and its subsets in predicting the presence and the extent of CAD in diabetic patients with stable angina pectoris. Additionally, our study determined the cut-off points of leukocytes and its subsets which can be most useful for predicting increased risk of severe CAD. Furthermore, we compared the relative predictive value of differential leukocyte counts and assessed which leukocyte subset was the most valuable marker for predicting the severity of CAD in patients with DM.

Nonetheless, there are several limitations in our study. Firstly, the relatively small sample size from a single center study is a limitation. Secondly, we did not combine leukocyte and its subsets count with other nonspecific inflammatory markers such as hs-CRP, fibrinogen and HbA1c to increase the predictive ability due to the small sample size. Moreover, although leukocyte and the severity of CAD in diabetic patients in the present study are significantly associated, the power was relatively small, and we failed to evaluate the predictive power of other leukocyte subsets such as eosinophils and basophils. Finally, we did not evaluate the predictive value of leukocytes and its subsets in our population. Thus, the data should be replicated in a study with larger sample size and long term follow up.

## Supporting Information

Table S1 **Pearson and Spearman correlation between leukocyte and its subsets with hs-CRP, Hemoglobin A1c and Gensini Score.** Data are presented as coefficient; p value; hs-CRP = high sensitivity C-reactive protein; HbA1c = Glycosylated hemoglobin A1c.
(DOC)
