# Supplementary material for: Relation of Leukocytes and Its Subsets Counts with the Severity of Stable Coronary Artery Disease in Patients with Diabetic Mellitus
Source: PLoS One. 2014 Mar 5;9(3):e90663. doi: 10.1371/journal.pone.0090663 (PMC3944194; doi:10.1371/journal.pone.0090663)
Supplement: Table S1 — Pearson and Spearman correlation between leukocyte and its subsets with hs-CRP, Hemoglobin A1c and Gensini Score. Data are presented as coefficient; p value; hs-CRP = high sensitivity C-reactive protein; HbA1c = Glycosylated hemoglobin A1c. (DOC) [file pone.0090663.s001.doc]

# Supporting Information

**Table S1.**

| Variables | Leukocyte | Neutrophil | Lymphocyte | Monocyte |
| --- | --- | --- | --- | --- |
| Pearson’ correlation |  |  |  |  |
| hs-CRP | 0.198; <0.000 | 0.277; <0.000 | -0.087; <0.093 | 0.155; <0.003 |
| HbA1c | 0.081; <0.118 | 0.105; <0.043 | 0.071; <0.172 | -0.159; <0.002 |
| Gensini Score | 0.154; <0.003 | 0.156; <0.003 | 0.050; <0.336 | 0.025; <0.636 |
| Spearman’ correlation |  |  |  |  |
| hs-CRP | 0.229; <0.000 | 0.266; <0.000 | -0.039; <0.450 | 0.137; <0.008 |
| HbA1c | 0.062; <0.231 | 0.089; <0.087 | 0.068; <0.190 | -0.191; <0.000 |
| Gensini Score | 0.168; <0.001 | 0.168; <0.001 | 0.019; <0.711 | 0.058; <0.264 |
